# Estimating the price elasticity of demand for cigarettes in South Africa using the Deaton approach

**DOI:** 10.1136/bmjopen-2020-046279

**Published:** 2021-12-16

**Authors:** Chengetai Dare, Micheal Kofi Boachie, Ernest Ngeh Tingum, S M Abdullah, Corné van Walbeek

**Affiliations:** 1 Research Unit on the Economics of Excisable Products (REEP), School of Economics, University of Cape Town, Rondebosch, South Africa; 2 SAMRC Centre for Health Economics and Decision Science -PRICELESS SA, Wits School of Public Health, Faculty of Health Sciences, University of the Witwatersrand, Parktown, Johannesburg, South Africa; 3 Department of Economics, University of Namibia, Windhoek, Khomas, Namibia; 4 Economics, University of Dhaka, Dhaka, Bangladesh; 5 Research and Development, ARK Foundation, Dhaka, Bangladesh

**Keywords:** health economics, public health, health policy, international health services, health services administration & management

## Abstract

**Objective:**

To estimate the price elasticity of demand for South Africa and thereby contribute to growing the evidence base of the likely impact of excise taxes on cigarette demand in low-income and middle-income countries.

**Methods:**

We employ the Deaton method, using wave 5 data from the South African National Income Dynamics Study, to estimate the cigarette price elasticity for South Africa. We used a sample of 6820 households.

**Results:**

Of the 6 820 households in the sample for which we had sufficient data, 1341 (19.7%) spent money on tobacco. The price elasticity of demand for cigarettes is estimated at −0.86 (95% CI −1.37 to −0.35), implying that the demand for cigarettes in South Africa declines by 8.6% for every 10% increase in price.

**Conclusion:**

The negative price elasticity estimate for South Africa indicates that increases in the excise tax are particularly effective in controlling cigarette consumption. However, given the presence of a significant illicit tobacco market in the country, it is important that authorities augment tax measures with strategies that curb the illicit trade in cigarettes.

**Strengths and limitations of this study**

- The study uses the Deaton approach, which is well-established internationally but novel in the South African context, to estimate the price elasticity of demand for cigarettes using nationally representative household survey data.
- The study demonstrates the efficacy of using the Deaton method in the estimation of price elasticities, and the fact that our estimates are in line with previous studies may encourage researchers in other countries to consider using this method for generating local evidence.
- Because time-series data are not available in many low-income and middle-income countries, this approach provides a credible alternative to time-series analysis for estimating the price and income elasticities of demand for tobacco products.
- A limitation is that the study used household-level estimates, which may not reflect individual-level estimates.

## Introduction

Tobacco consumption accounts for the deaths of more than eight million people annually across the world.^1 2^ Over 7 million of those deaths are the result of direct tobacco consumption, and around 1.2 million deaths are the result of exposure to secondhand smoke. Since more than 80% of tobacco users reside in low-income and middle-income countries (LMICs), the public health implications in those countries are considerably worse than in high-income countries.^1 3 4^

In Africa, South Africa has the fifth highest smoking prevalence, after the Republic of Congo, Lesotho, Sierra Leone and Namibia.^5 6^ Smoking prevalence among South African adults has been estimated to be around 19% of the adult population.^7 8^ Smoking is more prevalent among males (34%) than among females (6.9%).^7^ There are also racial differences in smoking patterns in South Africa, with whites (26%), Coloureds (people with mixed racial heritage, 41%) and Indians (24%) having a substantially higher smoking prevalence than Africans (16%).^9^

Like many other governments across the world, the South African government uses a variety of measures to reduce tobacco use. The strategies range from tax and price measures to non-price strategies such as limits on public smoking and strict controls on tobacco advertising. The government has drafted (but not yet passed) legislation that will, among other things, introduce pictorial health warnings and plain packaging, make all public areas 100% smoke-free, and regulate electronic nicotine delivery systems in the same way as tobacco products. In a controversial move, the country banned the sale of cigarettes for nearly 5 months during the COVID-19 lockdown in 2020.^10^

Illicit trade in cigarettes is a concern, independently estimated at about 30%–35% of the total market in 2017.^7 8^ At the time of writing (August 2021) the country had not ratified the Protocol to Eliminate the Illicit Trade in Tobacco Products. Despite a serious attempt by the South African Revenue Services to implement a Track and Trace system in 2019, the request for tenders was withdrawn in early 2020.

**Figure 1:**
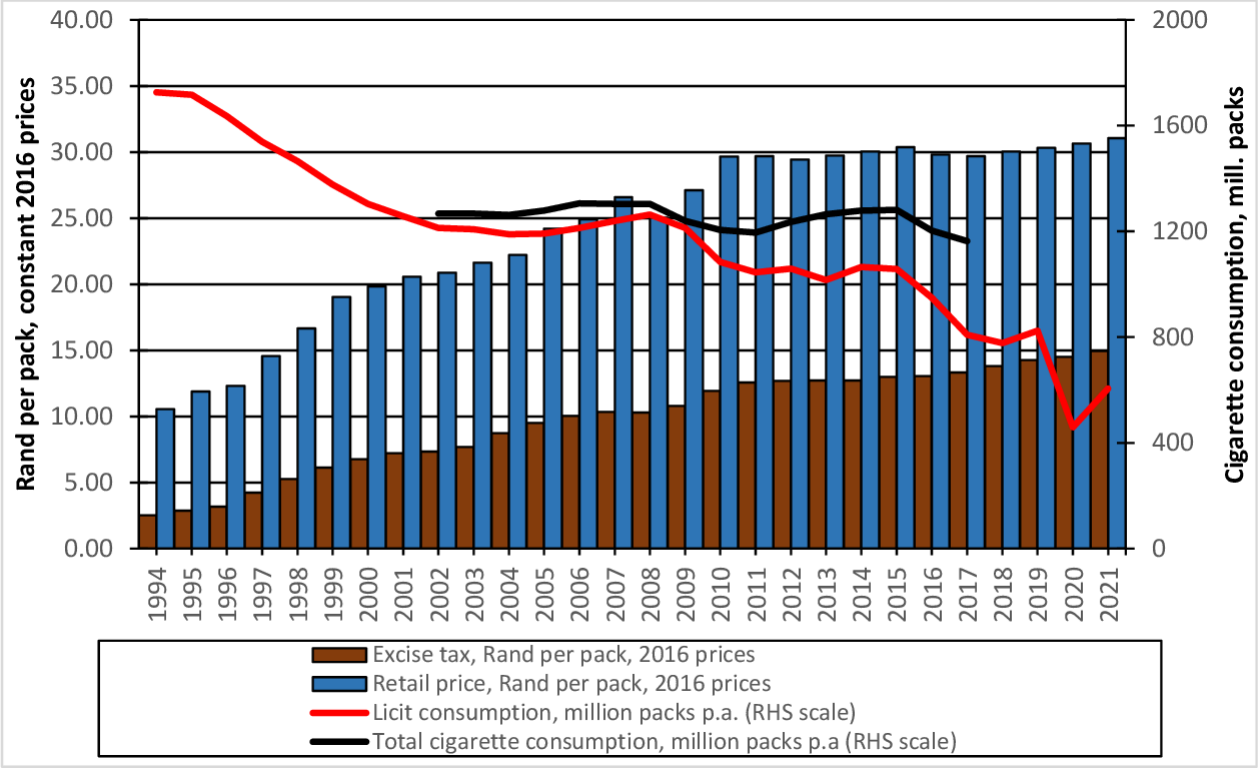
Trend of excise tax, price and consumption. Source: Republic of South Africa: Budget Reviews, numerous years. Statistics South Africa: Consumer price index. http://www.statssa.gov.za/publications/P0141/CPIHistory.pdf, and P0141 releases (numerous months).

Of the various tobacco control measures, excise tax increases are the single most important strategy for reducing tobacco consumption.^4^ An increase in the excise tax increases the retail price of tobacco products, making them less affordable, and reduces the demand for them. In South Africa, the excise tax has been levied as a uniform specific tax for at least a century, and the rate has been regularly adjusted. However, the tax burden remains considerably below the 75% recommended by WHO.^2^ For example, in the year 2020—as can be seen in figure 1—the average price of cigarettes was R30.66, and the specific tax was R14.52. Thus, the tax burden per package is approximately 47.4% (=14.52/30.66). The trend of excise tax, cigarette price, and consumption is depicted in figure 1. Total cigarette consumption figures are derived using Vellios *et al*’s estimates^8^ of the illicit market share.

As shown in figure 1, since 1994, both the excise tax and the retail prices have been increasing steadily, while cigarette consumption has been declining. These trends seem to confirm the generally understood notion that excise tax increases the retail price, which reduces consumption.^4^

The literature indicates that the demand for tobacco products is more price-sensitive in LMICs than in high-income countries, but nearly all studies find that the price elasticity falls in the inelastic range.^3 11 12^ In South Africa, a number of studies have estimated the price elasticity of cigarette demand. Initial estimates were mostly based on time-series analyses. For instance, Reekie,^13^ using consumption data for the period 1970–1989, found the price elasticity of cigarette to be −0.87. Van Walbeek,^14^ using 1970–1990 data from a variety of sources, estimated the long-run price elasticity to range between −0.53 and −1.52. Van der Merwe and Annett^15^ used data for 1970–1995 and found a value of −0.69, while Van Walbeek^16^ found a value of −0.6 using 1970–1998 annual data. In a later study, Van Walbeek^17^ looked at annual data from 1970 to 2003 and found the price elasticity to be −0.99. Boshoff^18^ used quarterly data for the period 1996–2006 and found the price elasticity of demand to lie between −0.5 and −0.9. Mukong and Tingum^19^ used longitudinal data drawn from the South African National Income and Dynamic Study (NIDS), and found a value of −0.43. Boachie and Ross^20^ used household-level data for six South African townships and found a value of −0.3, which is lower (in absolute terms) than previous estimates.

All previous studies, other than Mukong and Tingum^19^ and Boachie and Ross,^20^ were based on aggregated time-series data, making it impossible to account for individual-level characteristics in the effects of price changes on smoking patterns. We seek to add to the growing literature by estimating the price elasticity of demand for cigarettes using an alternative approach, first proposed by Deaton^21^ and extended in subsequent years.^22 23^

Previous studies in Uganda,^24^ Nigeria,^25^ India^26 27^ and other countries^28–31^ followed this method to obtain price elasticities using expenditure survey data. These studies found that the price elasticity for Uganda ranged between −0.26 and −0.33; for Nigeria between −0.49 and −0.63; and for India between −0.4 and −0.9. The price elasticity estimates for Ecuador and Papua New Guinea were found to be −0.87 and −1, respectively. All these estimates compare favourably with those for South Africa.

The Deaton approach is based on the theory of consumer behaviour, where households’ expenditure on each commodity reflects the quantity, quality and price of that commodity.^22 26 27^ These three dimensions differ between households. Thus, in the context of cigarette demand, a smoker not only chooses the quantity of cigarettes but also the characteristics of the cigarettes when making purchases. Following a price hike, smokers may change not only the quantity of cigarettes purchased, but may purchase a lower ‘quality’ brand of cigarettes. Using the Deaton approach, this paper provides a new perspective on the issue, and may add to the growing literature on estimating price elasticity of demand. Considering that the Deaton method relies on expenditure data, which are widely available in many LMICs, this paper seeks to demonstrate the efficacy of using this approach in the estimation of price elasticities. This method may be useful in settings where price elasticity estimations are hindered by the unavailability of aggregate demand and market price data. In line with previous studies, we expect to find a negative and less-than-unit price elasticity estimate.

## Methodology

### Data

The study uses wave 5 data from the South African NIDS^9^ to estimate cigarette price elasticity. NIDS is a face-to-face longitudinal survey which tracks income, labour market participation and other household characteristics since 2008. It is nationally representative, covering all nine South African provinces and uses a multistage stratified sampling technique. Demographic and socioeconomic characteristics (such as race, age, gender, level of education and employment status) are also gathered. The NIDS survey is conducted in waves, the latest being wave 5 (collected in 2017). The wave 5 dataset comprises 10 647 households. Unlike previous waves, wave 5 captured information on the quantity of cigarettes purchased and the amount spent by individuals for their most recent purchase.^9^ From these data, we were able to calculate the unit value paid per pack of cigarettes (or equivalent). In cases where the resultant unit values were meaningless, we cleaned the data following the conventions described in van der Zee *et al*.^7^ The cleaned data gives a sample of 6820 households, of which 1341 (19.7%) reported positive tobacco consumption. Of the total number of household heads, 57.1% are females. The descriptive statistics are shown in table 1.

**Table 1:** Descriptive statistics

Variable	N (or percentage of total sample)
No of households	6820
Households reporting positive consumption	1341
No of clusters	385
Average unit value (Rand)	1.50
Average quantity purchased in the month (cigarette sticks)	97.0
Average household size (number of individuals)	4.2
Average age of household head (years)	41.2
Percentage of adults in the household	70.0
Average cigarette share in household expenditure (percentage)	2.0
Percentage of males in the household	60.0
Race (percentage)	
African	78.6
Coloured	12.9
Asian/Indian	1.9
White	6.6
Highest educational level in household (percentage)	
No school at all	2.5
Up to primary	41.4
Matric/secondary school	22.3
College/university/tertiary education	33.8
Gender of household head (percentage)	
Male	42.9
Female	57.1
Employment status of household head (percentage)	
Employed	60.2

### Empirical estimation

The Deaton approach follows several steps which have been detailed elsewhere in the literature.^22 24 27 32^ We began our analysis by obtaining unit values (expenditure divided by quantity) and budget shares for each household in each cluster. The budget share was obtained by dividing the monthly expenditure on cigarettes by the total monthly household expenditure (budget). We then tested for spatial variation in the unit values using the analysis of variance (ANOVA) test. The null hypothesis of no spatial variation in the unit values was rejected. Afterwards, the within-cluster regressions were estimated. The within-cluster estimates are obtained as the means from the budget shares and unit value regressions:

(1) $$lnuv_{ic}=\propto^{1}+\beta^{1}lnx_{ic}+\gamma^{1}Z_{ic}+\varphi ln\pi_{c}+\mu_{ic}^{1}$$

(2) $$w_{ic}=\propto^{0}+\beta^{0}lnx_{ic}+\delta Z_{ic}+\emptyset ln\pi_{c}+\left(f_{c}+\mu_{ic}^{0}\right)$$

Equations 1 and 2 represent, respectively, the unit value and budget share regressions for household *i* living in cluster *c*. In the above equations, the log of the unit value, $lnuv_{ic}$, is a function of household expenditure ($lnx_{ic}$) and a vector of household characteristics ($Z_{ic}$). The household characteristics include the highest educational level of a household member, gender of the household head, age of the household head, employment status of the household head, race, proportion of adults in the household, and proportion of males in the household. These variables are introduced to purge the unit values of the household-specific effects that are associated with the quality effects, so as to enable a consistent estimation of price elasticities.^22 27^ As such, the unit value equation allows us to check for the presence of quality effects. A positive and statistically significant relationship between household expenditure and unit values (ie, $\beta^{1}$), indicates the presence of quality effects, i.e. more affluent households purchase more expensive, higher quality cigarettes. $ln\pi_{c}$ are unobserved cigarette prices and, consequently, equations 1 and 2 are estimated without them but their coefficients are recovered through the formulas contained in equations 7 and 8.^32^

In the second equation, $w_{ic}$ is the budget share of cigarettes in the total budget of household *i* living in cluster *c*. The budget share of the household is taken to be a linear function of the logarithm of total household expenditure, $x_{ic}$, a vector of household characteristics, $Z_{ic}$, and the logarithm of unobserved cigarette prices ($ln\pi_{c}$). As in equation 1, the variables in $Z_{ic}$ are introduced only to remove household-specific effects from the budget shares. $f_{c}$ is the cluster fixed effect, which is assumed to be uncorrelated with price. $u_{ic}^{0}$ and $u_{ic}^{1}$ are regression error terms. φ is the elasticity of the unit value with respect to price, while $\beta^{0}$ is the total expenditure elasticity with respect to the budget share spent on cigarettes.^22 33^

The next stage involves removing the effects of household expenditure and household characteristics from the household-level demand and unit values and then averaging across clusters.^32^ This step requires the following equations:

(3) $$\begin{array}{l} {\hat{y}}_{c}^{1}=\frac{1}{n_{c}}\sum_{i=1}^{n_{c}}\left(lnuv_{ic}-{\hat{\beta }}^{1}lnx_{ic}-\hat{y}Z_{ic}\right) \end{array}$$

(4) $$\begin{array}{l} {\hat{y}}_{c}^{2}=\frac{1}{n_{c}}\sum_{i=1}^{n_{c}}\left(w_{ic}-{\hat{\beta }}^{0}lnx_{ic}-\hat{\delta }Z_{ic}\right) \end{array}$$

where $n_{c}$ is the number of households in cluster *c*. ${\hat{y}}_{c}^{1}$ and ${\hat{y}}_{c}^{2}$ are respectively the estimates of average unit value and cluster average demand for cluster *c* after removing the effects of expenditure and household characteristics. Given that the underlying condition for the Deaton method is that prices vary between clusters (and not within clusters), the price elasticity of demand can only be obtained at cluster level. This requires regressing cluster-level demand (${\hat{y}}_{c}^{2}$) on cluster-level unit values (${\hat{y}}_{c}^{1}$). Alternatively, the coefficient on ${\hat{y}}_{c}^{1}$ can be obtained by dividing the covariance between ${\hat{y}}_{c}^{2}$ and ${\hat{y}}_{c}^{1}$ by the variance of ${\hat{y}}_{c}^{1}$ as follows:

(5) $$\hat{\rho }=\frac{Cov\left({\hat{y}}_{c}^{2},{\hat{y}}_{c}^{1}\right)-\frac{{\hat{\sigma }}^{12}}{n_{c}}}{Var\left({\hat{y}}_{c}^{1}\right)-\frac{{\hat{\sigma }}^{11}}{n_{c}+}}$$

where $\hat{\rho }$ is the coefficient on ${\hat{y}}_{c}^{1}$; $n_{c}^{+}$ is the average number of households in a cluster reporting positive expenditures on cigarettes; $n_{c}$ is the average number of households in a cluster, irrespective of smoking status; ${\hat{\sigma }}^{12}$ is the estimate of the covariance of the errors in equations (1) and (2); and ${\hat{\sigma }}^{11}$ is the variance in the errors of equation (1). The error terms correct the price elasticity estimates (equation 6) for measurement errors.

Quality correction formulas are then applied to obtain the estimate of the price elasticity, ${\hat{\varepsilon }}_{p}$, as follows:

(5) $${\hat{\varepsilon }}_{p}=\left(\frac{\hat{\emptyset }}{{\bar{w}}_{c}}\right)-\hat{\varphi }$$

where ${\overset{-}{w}}_{c}$ is the cluster-level average share of total household expenditure on cigarettes. $\hat{\varphi }$ and $\hat{\emptyset }$ are estimates of the coefficients on the unobserved price terms in equations (1) and (2), respectively. They are recovered as follows:

(7) $$\hat{\varphi }=1-\frac{{\hat{\beta }}_{c}^{1}\left({\bar{w}}_{c}-\hat{\emptyset }\right)}{{\hat{\beta }}_{c}^{0}+{\bar{w}}_{c}}$$

(8) $$\hat{\emptyset }=\frac{\hat{\rho }}{1+\left({\bar{w}}_{c}-\hat{\rho }\right)\hat{\vartheta }}$$

(9) $$\hat{\vartheta }=\frac{{\hat{\beta }}_{c}^{1}}{{\hat{\beta }}_{c}^{0}+\bar{w}\left(1-{\hat{\beta }}_{c}^{1}\right)}$$

Deaton^22 24 26 33^ proposes the following formula for obtaining the estimate of the expenditure (income) elasticity of demand, ${\hat{\varepsilon }}_{x}$ :

(10) $${\hat{\varepsilon }}_{x}=1+\left(\frac{{\hat{\beta }}^{0}}{\bar{w}}\right)-{\hat{\beta }}^{1}$$

where $\overset{-}{w}$ is the average share of total household expenditure dedicated to cigarettes in the sample.

### Patients and public involvement

Patients and the public were not involved in this study.

## Results

**Figure 2:**
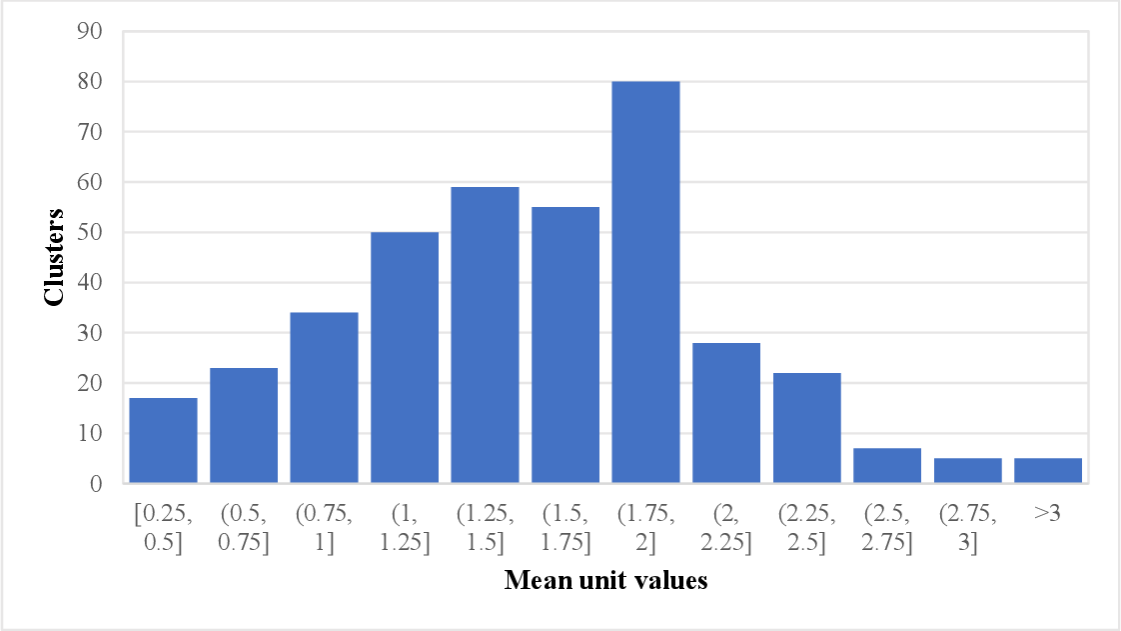
Distribution of unit values across clusters.

The key identifying condition for the Deaton method is that prices vary geographically. Using the harmonic mean to calculate cluster size, we found that, based on 6820 households, there are about 13 households in a cluster. Figure 2 depicts the frequency of the unit value across clusters.

We employed ANOVA techniques to test whether there is spatial variation in prices, proxied by unit values. The results of the ANOVA estimation exercise are depicted in table 2.

**Table 2:** Testing the spatial variation hypothesis

F statistic	P value	R^2^	N
1.89	0.000	0.43	385

The F statistic and the p value associated with the null hypothesis indicate that there is spatial variation in the unit values, as the null hypothesis of no spatial variation is rejected (table 2). The R^2^ of 0.43 means that 43% of the variation in prices takes place between clusters.

Table 3 presents the estimated coefficients from both the unit value and budget share regressions (equations 1 and 2). The regressions account for cluster effects.

**Table 3:** Results of the unit value and budget share regressions

Variables	Unit value regression	Budget share regression
Log household expenditure	0.107*** (0.026)	−0.001*** (0.001)
Log household size	−0.056 (0.045)	0.002*** (0.000)
Share of adults in the household	−0.15 (0.107)	0.004*** (0.001)
Share of males in the household	0.12 (0.025)	0.002*** (0.000)
African	0.000	0.000
Coloured	0.112 (0.094)	0.003*** (0.001)
Asian/Indian	0.527** (0.219)	0.003 (0.002)
White	0.321** (0.156)	0.002** (0.001)
No schooling at all	0.000	0.000
Up to primary level	−0.262 (0.246)	0.001 (0.001)
Secondary/matric level	−0.192 (0.249)	0.000 (0.001)
Tertiary	−0.213 (0.249)	0.000 (0.001)
Male	0.000	0.000
Female	−0.031 (0.037)	−0.002 (0.003)
Age	−0.006 (0.006)	0.000*** (0.000)
Not working	0.000	0.000
Working	0.014 (0.040)	0.001 (0.001)
Fixed effects	Yes	Yes
Constant	−0.060 (0.344)	0.014*** (0.004)
Number of households	1341	6820
R^2^	0.45	0.18

*** p<0.01, ** p<0.05, * p<0.10

Unit values increase by 0.11% for every 1% increase in household expenditure, suggesting that richer households have higher unit values (table 3). The positive and statistically significant relationship between total household expenditure and unit values, after accounting for household characteristics, indicates the presence of quality effects. Coloureds, Asians and whites all have higher unit values than black Africans.

There is a negative and statistically significant relationship between total household expenditure and the share of household budget allocated to cigarettes. The cigarette budget share declines by 0.01% as total household expenditure increases by 1%, indicating that cigarette purchases are regressive. We also found that the budget share on cigarettes increases as the size of household increases. Similarly, households increase their budget share on cigarettes as the proportion of adults in the household increases. Predictably, the budget share on cigarettes increases as the male-to-female ratio in the household increases. Since the focus is not on control variables, we are not discussing all of them here.

To obtain the unconditional price elasticity of quantity demanded, we used the information in table 3, along with parameters obtained from equation 3–9. The parameters are shown in table 4.

**Table 4:** Key parameters

${\hat{\beta }}^{1}$	0.1067
${\hat{\beta }}^{0}$	−0.0013
$\overset{-}{w}$	0.0036
$Cov\left({\hat{y}}_{c}^{1},{\hat{y}}_{c}^{2}\right)$	−0.0001
$Var\left({\hat{y}}_{c}^{1}\right)$	0.2019
${\hat{\sigma }}^{11}$	0.2714
${\hat{\sigma }}^{12}$	0.0001
$n_{c}$	5.0544
$n_{c}^{+}$	2.0840
${\hat{\beta }}_{c}^{1}$	0.1314
${\hat{\beta }}_{c}^{0}$	−0.0015
${\overset{-}{w}}_{c}$	0.0025
$\hat{\rho }$	−0.0011
$\hat{\varphi }$	0.5949
$\hat{\emptyset }$	−0.0006
$\hat{\vartheta }$	192.0178

The estimates of the unconditional price elasticity of demand for cigarettes are obtained from equation 6, while the income elasticities are obtained from equation 10, based on the first stage regressions (equations 1 and 2). The estimates are depicted in table 5.

**Table 5:** Estimate of price (and income) elasticity of demand for cigarettes in South Africa

	Price elasticity	Expenditure (income) elasticity
Elasticity estimate	−0. 857***	0.544***
Bootstrap SE	0.260	0.077
95% CI	−1.368 to −0.347	0.392 to 0.696

p<0.05, **p<0.01, ***p<0.001.

The price elasticity of demand for cigarettes is −0.86 (95% CI −1.37 to −0.35), while the income elasticity of demand is 0.54 (95% CI 0.392 to 0.696). The price and income elasticities of demand are bootstrapped using 1000 replications to obtain the standard errors and the associated CIs.

## Discussion

Increasing the excise tax is one of the most effective tobacco-control tools and has been used extensively in South Africa. The excise tax typically increases the retail price of cigarettes,^34^ thereby reducing the demand for cigarettes. However, the effectiveness of the excise tax depends crucially on the responsiveness of demand to price changes. Using household-level data, this paper sought to estimate the price elasticity of demand for cigarettes in South Africa. The unconditional price elasticity of demand is estimated to be −0.86 (95% CI −1.37 to −0.35), implying that a 10% rise in the price is associated with an 8.6% reduction in cigarette demand. Thus, as expected, the price elasticity of demand for cigarette for South Africa is negative and inelastic. This result aligns well with most previous studies.^13–15 17 18^ However, the result is different from those found by Boachie and Ross,^20^ and by Mukong and Tingum.^19^ This is because the survey data used by Boachie and Ross capture only small parts of the South African society. Thus, the survey is not a representative national survey. In the case of Mukong and Tingum, they use actual price data at the regional level and not unit values. However, a consistent finding across all these studies is that the price elasticity of cigarette demand for South Africa is negative and inelastic. Considering the current relatively low excise tax burden, the government should consider increasing the excise tax burden in line with the recommendations of the WHO.^35^

The income elasticity (measured by the expenditure elasticity) shows that a 1% increase in household income is associated with a 0.54% increase in cigarette consumption. The limitation of the income elasticity is that it does not capture the actual income of the household since it is measured by expenditure.

Studies that model the impact of an excise tax increase have shown that, for all realistic values of the price elasticity, a tax-induced increase in the price of cigarettes reduces tobacco use and increases government revenue.^36^ Within this win-win scenario, there is a trade-off, however. For a given tax increase, a relatively lower (in absolute terms) price elasticity yields relatively more additional revenue but a lower reduction in consumption, while a relatively higher price elasticity yields relatively less additional revenue but a greater reduction in consumption. Given that the price elasticity in South Africa is negative and inelastic, an increase in the excise tax will not only reduce the demand for cigarettes but will be effective in increasing revenue.^36 37^

However, the effectiveness of an excise tax could be reduced by the presence of a significant illicit cigarette market, which has been increasing in South Africa since 2010, and comprised about 30%–35% of the total market when the NIDS wave 5 survey was conducted in 2017.^7 8 38^ The 20-week sales ban in 2020, imposed to reduce the pressure on South Africa’s health sector, has probably entrenched the illicit market further.^39^ The illicit market provides an alternative to the official market. In an environment where illicit cigarettes are easily accessible, and where the illicit market is already well-established, price increases in the official market could drive smokers into the alternative market. Illicit trade in cigarettes is often an indication of weak administrative and/or enforcement measures by the authorities overseeing the tobacco market. It is therefore important that the government not only increases the excise tax, but also implements strong and effective controls to curb the illicit tobacco market. Otherwise, the tobacco industry, illicit traders and smokers may take advantage of the enforcement loopholes and substitute licit cigarettes with illicit ones, to avoid or evade the tax, thereby reducing the effectiveness of the excise tax.

Although the estimates presented in this study are useful guides for devising suitable tobacco-tax policy measures, these findings have limitations. For instance, the Deaton method is designed for household data. For this study we aggregated the individual-level purchasing behaviours to the household, rather than looking at the household per se. As such, this analysis does not focus on individuals, but rather on households. Household-level estimates may not reflect individual-level estimates.

## Conclusion

This study used the Deaton method to estimate the price elasticity of demand for cigarettes in South Africa, using wave 5 data from NIDS, a nationally representative household survey for South Africa. The Deaton method relies on expenditure data, which are widely available across the African continent. The paper sought to demonstrate the efficacy of using the Deaton method in the estimation of price elasticities, especially in settings where such estimations are hindered by the unavailability of aggregate demand and market price data. This, together with the fact that our estimates are in line with previous estimates for South Africa, will hopefully encourage other researchers to consider employing this method for generating local evidence.

The price elasticity of demand for cigarettes is estimated at −0.86 (95% CI −1.37 to −0.35), implying that the demand for cigarettes in South Africa declines by 8.6% for every 10% increase in price. Our results show that increasing the excise tax is particularly effective at reducing tobacco demand in South Africa. Considering the current relatively low excise tax burden, the government should consider increasing the excise tax burden in line with the recommendations of the WHO.^35^ However, given the presence of a significant illicit tobacco market in the country, it is important that authorities augment tax measures with strategies that curb the illicit trade in cigarettes.

## Data Availability

Data are available in a public, open access repository. Data are publicly available on http://www.nids.uct.ac.za/nids-data/data-access.
